# Supplementary material for: The Systems Biology Research Tool: evolvable open-source software
Source: BMC Syst Biol. 2008 Jun 29;2:55. doi: 10.1186/1752-0509-2-55 (PMC2446383; doi:10.1186/1752-0509-2-55)
Supplement: Additional file 1 — SBRT Archive. An archive of the current version of the Systems Biology Research Tool. [file 1752-0509-2-55-S1.zip › sbrt-1.4.0/doc/developers_guide/api/sbrt/shell/io/fba/SfoofSource.html]

SfoofSource


|  |  |  |
| --- | --- | --- |
| **Overview** \| **Package** \| **Class** \| **Use** \| **Tree** \| **Deprecated** \| **Index** \| **Help** | |  |
| **PREV CLASS**   NEXT CLASS | **FRAMES**    **NO FRAMES**     **All Classes** |  |
| SUMMARY: NESTED \| FIELD \| CONSTR \| METHOD | DETAIL: FIELD \| CONSTR \| METHOD |  |


---


## sbrt.shell.io.fba Class SfoofSource

```
java.lang.Object
  sbrt.shell.io.fba.SfoofSource
```

**All Implemented Interfaces:**: java.util.Iterator<SerFbaOptOutputFile>

---

``` public class SfoofSource extends java.lang.Object implements java.util.Iterator<SerFbaOptOutputFile> ```

This class is used to provide a source of serialized
FBA optimization output files.

**Author:**
:   This class was written and documented by
    Jeremiah Wright while in the Wagner lab.

---

**Constructor Summary**

`SfoofSource(java.util.Iterator<java.lang.String> fileNameSource,
FileFormat fileFormat,
java.util.Set<java.lang.String> filterKeys,
FbaOptFileLineFormat fbaFormat)`
  
          Constructs a new source of serialized
FBA optimization output files.

`SfoofSource(java.util.Iterator<java.lang.String> fileNameSource,
java.lang.String fileNameSourceName,
FileFormat fileFormat,
java.util.Set<java.lang.String> filterKeys,
FbaOptFileLineFormat fbaFormat)`
  
          Constructs a new source of serialized
FBA optimization output files.


| **Method Summary** | |
| --- | --- |
| `FileFormat` | `getFileFormat()`             Returns the file format used to create files of type `SerFbaOptOutputFile`. |
| `boolean` | `hasNext()`             Indicates if this iterator can return another file using the `next()` method . |
| `SerFbaOptOutputFile` | `next()`             Returns the next column-oriented map output file with the next name returned by the file name source. |
| `void` | `remove()`             Throws `UnsupportedOperationException`. |

**Methods inherited from class java.lang.Object**

`clone, equals, finalize, getClass, hashCode, notify, notifyAll, toString, wait, wait, wait`

**Constructor Detail**

### SfoofSource

```
public SfoofSource(java.util.Iterator<java.lang.String> fileNameSource,
                   FileFormat fileFormat,
                   java.util.Set<java.lang.String> filterKeys,
                   FbaOptFileLineFormat fbaFormat)
```

:   Constructs a new source of serialized
    FBA optimization output files.

    **Parameters:**: `fileNameSource` - the file name source.: `fileFormat` - the file format used to write files.: `filterKeys` - a set of valid keys that will appear in the files produced by this source. This set can consist of reaction names and the objective function header.: `fbaFormat` - the map format used to supply a header object for objective values. **Throws:**: `java.lang.NullPointerException` - if any argument except `filterKeys` is `null`.

---


### SfoofSource

```
public SfoofSource(java.util.Iterator<java.lang.String> fileNameSource,
                   java.lang.String fileNameSourceName,
                   FileFormat fileFormat,
                   java.util.Set<java.lang.String> filterKeys,
                   FbaOptFileLineFormat fbaFormat)
```

:   Constructs a new source of serialized
    FBA optimization output files.

    **Parameters:**: `fileNameSource` - the file name source.: `fileNameSourceName` - the name of the file name source.: `fileFormat` - the file format used to write files.: `filterKeys` - a set of valid keys that will appear in the files produced by this source. This set can consist of reaction names and the objective function header.: `fbaFormat` - the map format used to supply a header object for objective values. **Throws:**: `java.lang.NullPointerException` - if any argument except `filterKeys` is `null`.


**Method Detail**

### getFileFormat

```
public FileFormat getFileFormat()
```

:   Returns the file format used to create files of
    type `SerFbaOptOutputFile`.

    :   **Returns:**: the file format used to create files of type `SerFbaOptOutputFile`.

---


### hasNext

```
public boolean hasNext()
```

:   Indicates if this iterator can return another file using the
    `next()` method
    .

    :   **Specified by:**: `hasNext` in interface `java.util.Iterator<SerFbaOptOutputFile>`
    :   **Returns:**: `true` if this iterator can return another file; `false` otherwise.

---


### next

```
public SerFbaOptOutputFile next()
```

:   Returns the next column-oriented map output file with the next
    name returned by the file name source.

    :   **Specified by:**: `next` in interface `java.util.Iterator<SerFbaOptOutputFile>`
    :   **Returns:**: the next column-oriented map output file.

---


### remove

```
public void remove()
```

:   Throws `UnsupportedOperationException`.

    :   **Specified by:**: `remove` in interface `java.util.Iterator<SerFbaOptOutputFile>`
    :   **Throws:**: `java.lang.UnsupportedOperationException`


---


|  |  |  |
| --- | --- | --- |
| **Overview** \| **Package** \| **Class** \| **Use** \| **Tree** \| **Deprecated** \| **Index** \| **Help** | |  |
| **PREV CLASS**   NEXT CLASS | **FRAMES**    **NO FRAMES**     **All Classes** |  |
| SUMMARY: NESTED \| FIELD \| CONSTR \| METHOD | DETAIL: FIELD \| CONSTR \| METHOD |  |


---
